# Medial Meniscus Posterior Root Reconstruction and Open-Wedge High-Tibial Osteotomy for Medial Meniscus Posterior Root Tear With Varus Knee Alignment: A Retrospective Study on Short-Term Outcomes

**DOI:** 10.7759/cureus.57170

**Published:** 2024-03-29

**Authors:** Hiroki Okamura, Hiroki Ishikawa, Takuya Ohno, Shogo Fujita, Shota Yamamoto, Shigeo Yamakami, Kei Nagasaki, Yoshifumi Kudo

**Affiliations:** 1 Department of Orthopaedic Surgery, Nihon Koukan Hospital, Kanagawa, JPN; 2 Department of Orthopaedic Surgery, Showa University School of Medicine, Tokyo, JPN

**Keywords:** meniscus tear, knee, osteoarthritis, open-wedge high-tibial osteotomy, medial meniscus posterior root reconstruction, medial meniscus posterior root tear

## Abstract

Purpose: We describe 13 cases of medial meniscus posterior root tear (MMPRT) with varus knee alignment treated with medial meniscus posterior root reconstruction (MMPR-R) and open-wedge high-tibial osteotomy (OWHTO) to identify an optimal MMPRT treatment.

Methods: We retrospectively reviewed 13 patients (mean age: 66.3 ± 8.0 years) who underwent MMPR-R and OWHTO. The Knee Injury and Osteoarthritis Outcome Score (KOOS), femorotibial angle (FTA), percentage mechanical axis (%MA) on radiography, and medial meniscus extrusion (MME) on magnetic resonance imaging (MRI) between the preoperative period and last follow-up were compared. Moreover, meniscus healing status and the International Cartilage Repair Society (ICRS) classification of the medial femoral condyle and medial tibial plateau on arthroscopy between the initial surgery and second-look arthroscopy were compared.

Results: The mean follow-up duration was 12.8 ± 2.2 months. At the last follow-up, the KOOS significantly improved (P < 0.01). Based on the FTA and %MA, the varus alignment was predominantly corrected at the last follow-up (P < 0.01). The MME was increased in nine (62.9%) patients, and the mean MME significantly increased at the last follow-up (P = 0.04). Second-look arthroscopy revealed improvements in the ICRS grade for the medial femoral condyle and medial tibial plateau in six (46.2%) patients. However, the results did not significantly differ. Regarding meniscus healing, four (30.8%) patients presented with complete healing, eight (57.1%) with partial healing, and one (7.7%) with failed healing.

Conclusions: The MMPRT with varus knee alignment significantly improved with MMPR-R and OWHTO. However, the MME and meniscus healing were unsatisfactory.

## Introduction

A medial meniscus posterior root tear (MMPRT) is a radial tear at < 10 mm from the posterior root attachment of the medial meniscus. The disruption of hoop action interrupts the kinematics of the knee joint [1, 2]. Hence, MMPRT triggers the rapid progression of knee osteoarthritis and subchondral insufficiency fracture of the knee [3, 4]; hence, appropriate treatment is important.

The treatment options for MMPRT include partial meniscectomy, conservative treatment, and meniscus repair [5-9]. In particular, suture anchor repair and pull-out suturing are widely used for meniscus repair, and they have good clinical outcomes [5, 6, 9]. However, some reports have shown that after these procedures, knee osteoarthritis progresses radiographically and arthroscopically. Therefore, the suitable treatment options remain controversial [5, 6, 9]. Li et al. reported that treatment by suturing a degenerated meniscus and implanting it into the bone tunnel is debatable as to the healing of the meniscus [10]. Therefore, in recent years, some studies have used medial meniscus posterior root reconstruction (MMPR-R) with autologous gracilis tendons. They reported that this technique has good outcomes [10-12]. Compared with meniscectomy or meniscus repair, MMPR-R is useful in reconstructing the original physiological properties of the meniscus and is beneficial for the functional recovery of the knee [11, 13]. Further, open-wedge high-tibial osteotomy (OWHTO) is recommended for MMPRT with > 4°-5°varus knee alignment, regardless of meniscus repair [14, 15]. A good meniscus healing rate can be achieved if meniscus repair and OWHTO are performed simultaneously [14]. Recently, Ohno et al. reported the first case of MMPRT with varus knee alignment treated with MMPR-R and OWHTO [12].

The current study aimed to describe 13 cases of MMPRT with varus knee alignment treated with MMPR-R and OWHTO.

## Materials and methods

Patient selection and data collection

This was a retrospective, cross-sectional study conducted at the Nihon Koukan Hospital, Kanagawa, Japan. The hospital's ethics committee issued approval (approval number: 202316). The medical records of 44 knees that underwent MMPR-R and OWHTO for MMPRT between April 2021 and December 2022 were reviewed. Intraoperative arthroscopic video was used to confirm MMPRT and other intraarticular lesions. All patients were diagnosed with MMPRTs on magnetic resonance imaging (MRI). This procedure was indicated for patients with MMPRT who presented with medial to popliteal pain, grade ≤ 2 osteoarthritis based on the Kellgren-Lawrence (K-L) system, grades ≤ 3 and 4 cartilage defects based on the International Cartilage Repair Society (ICRS) classification, which could be repaired with microfracture, and varus alignment of the lower extremity, which could be corrected with OWHTO. Patients who received treatment with other surgical techniques (MMPR-R and pull-out suturing and/or centralization with OWHTO) (29 knees), those with incomplete follow-up MRI measurements (one knee), and those without Knee Injury and Osteoarthritis Outcome Score (KOOS) (one knee) were excluded. Finally, 13 patients with MMPRT who underwent MMPR-R with OWHTO were included in this analysis. All patients underwent metal removal and a second examination after at least 12 months. None of the patients had a history of trauma (such as a fracture around the knee, ligament, and/or meniscal injury) or rheumatoid arthritis on the affected side. Data on the following parameters were recorded: age, sex, body mass index (BMI), affected side, radiographic measurements (femorotibial angle (FTA), posterior tibial slope (PTS), and percentage of mechanical axis (%MA)) before surgery and during the last follow-up, medial meniscus extrusion (MME) on MRI, KOOS before surgery and during the last follow-up, cartilage status based on the ICRS classification of the medial femoral condyle (MFC) and medial tibial plateau (MTP), and meniscus healing status at the index surgery and second-look arthroscopy.

Surgical technique and post-rehabilitation

**Figure 1:**
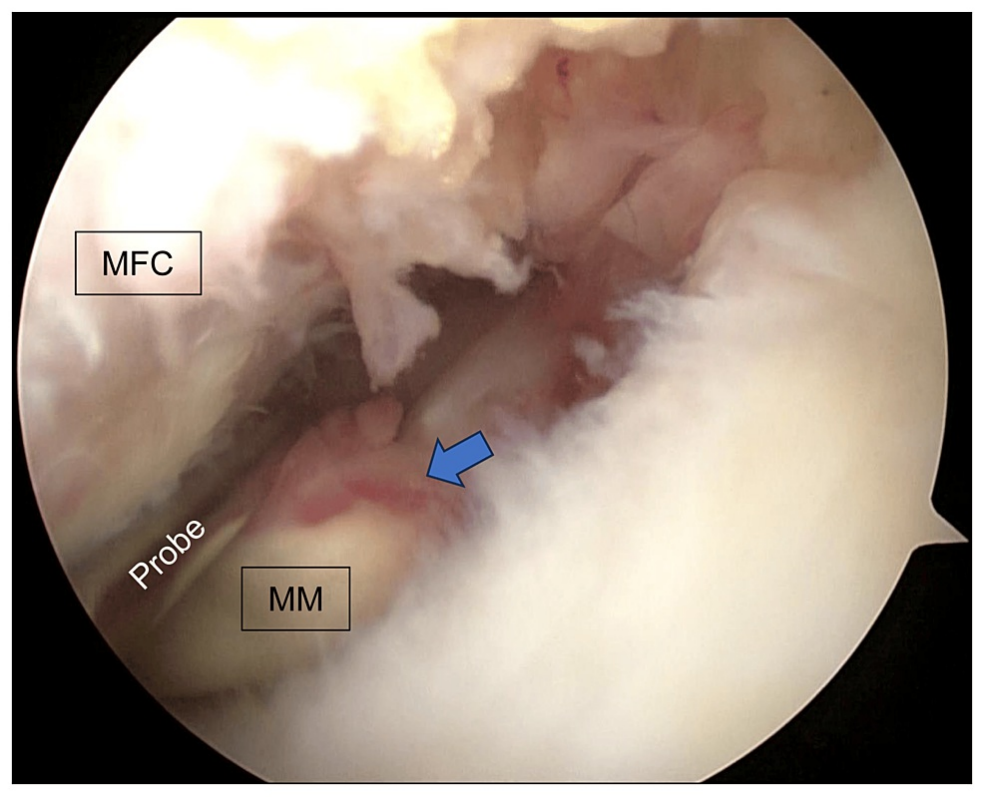
Diagnosis of MMPRT An arthroscopic image of the left knee; MMPRT was confirmed using a probe. MFC: medial femoral condyle; MM: medial meniscus; MMPRT: medial meniscus posterior root tear

The surgical procedure for MMPR-R with OWHTO was as follows: First, MMPRT was confirmed using a probe (Figure 1).

**Figure 2:**
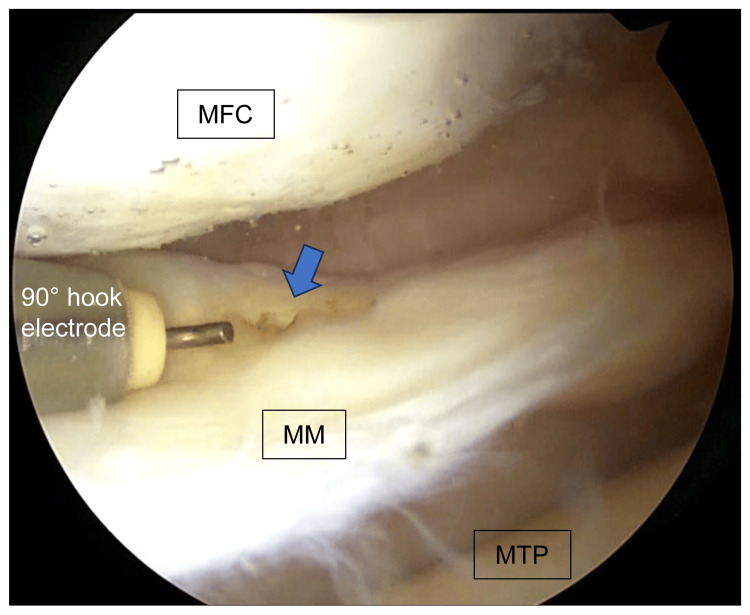
Creation of a soft tissue tunnel An arthroscopic image of the left knee; a soft tissue tunnel was created at the MM periphery approximately 5 mm from the medial meniscus posterior root tear edge (blue arrow) using a 3.5-mm 90°electrode (Mitek VAPR 3 System). MFC: medial femoral condyle; MM: medial meniscus

Second, an oblique skin incision of approximately 7 cm was made on the medial side of the tibia, and the gracilis was harvested. The harvested gracilis was double-folded and formed into an implanted tendon. Third, a soft tissue tunnel was created at the medial meniscus periphery approximately 5 mm from the MMPRT edge using a 3.5-mm 90°electrode (Mitek VAPR 3 System, Depuy Mitek, Raynham, MA) and 60° hooked rotary scissors (ACUFEX, Smith & Nephew, Hertfordshire, UK) (Figure 2).

**Figure 3:**
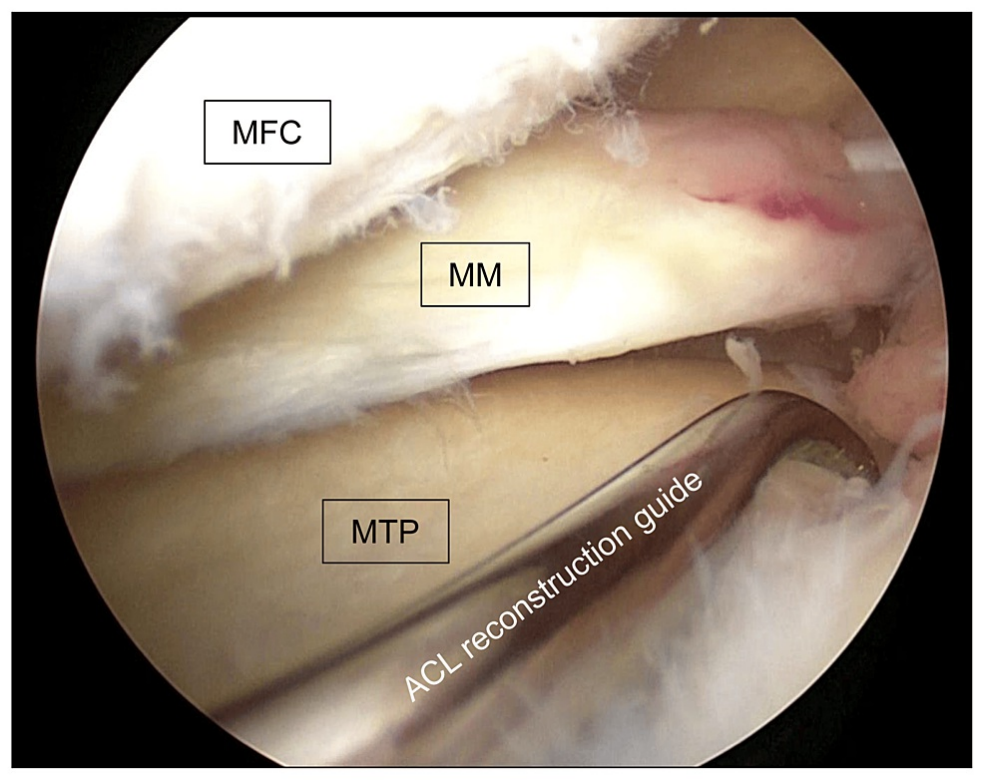
Insertion of the guide pin into the tibial tunnel An arthroscopic image of the left knee; a guide pin was inserted into the tibial tunnel using an anterior cruciate ligament reconstruction guide (3M). MFC: medial femoral condyle; MM: medial meniscus; MTP: medial tibial plateau

Fourth, a guide pin was inserted into the tibial tunnel using an anterior cruciate ligament reconstruction guide (3M, Saint Paul, MN) (Figure 3).

**Figure 4:**
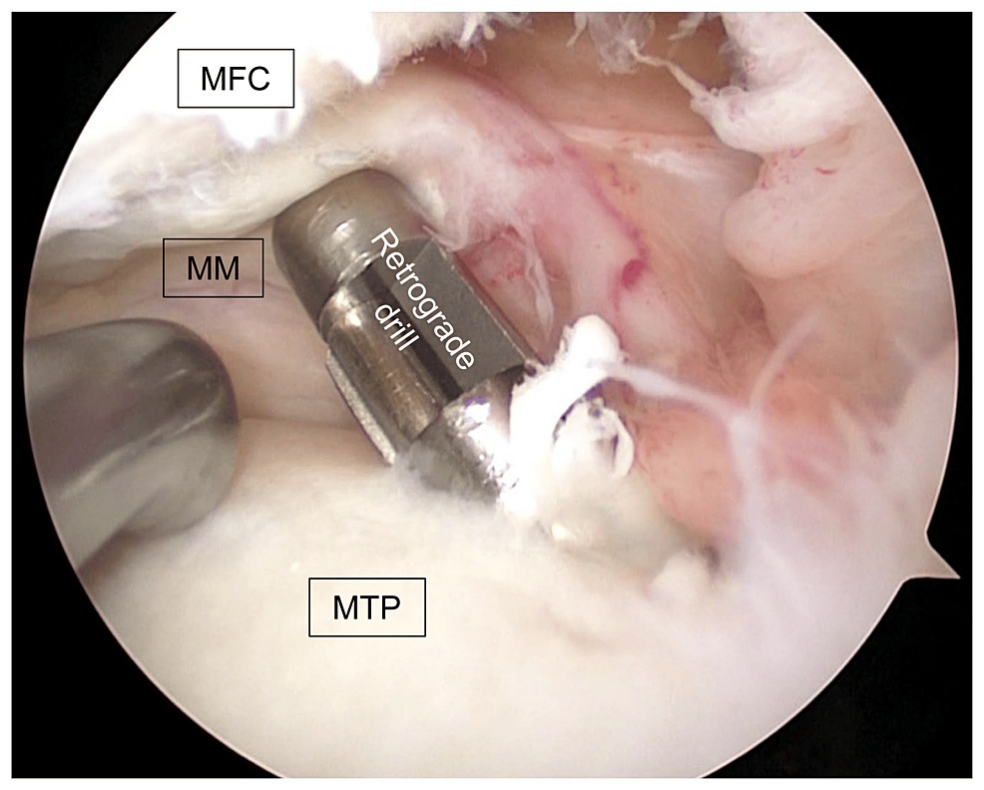
Creation of a tibial tunnel An arthroscopic image of the left knee; after creating a tibial tunnel with a 4.5-mm diameter drill via the guide pin, the articular surface was enlarged with a 6.0-mm retrograde drill (AI-drill). MFC: medial femoral condyle; MM: medial meniscus; MTP: medial tibial plateau

Fifth, after creating a tibial tunnel with a 4.5-mm diameter drill via the guide pin, the articular surface was enlarged with a 6.0-mm retrograde drill (AI-drill, AI-Medic, Tokyo, Japan) (Figure 4).

**Figure 5:**
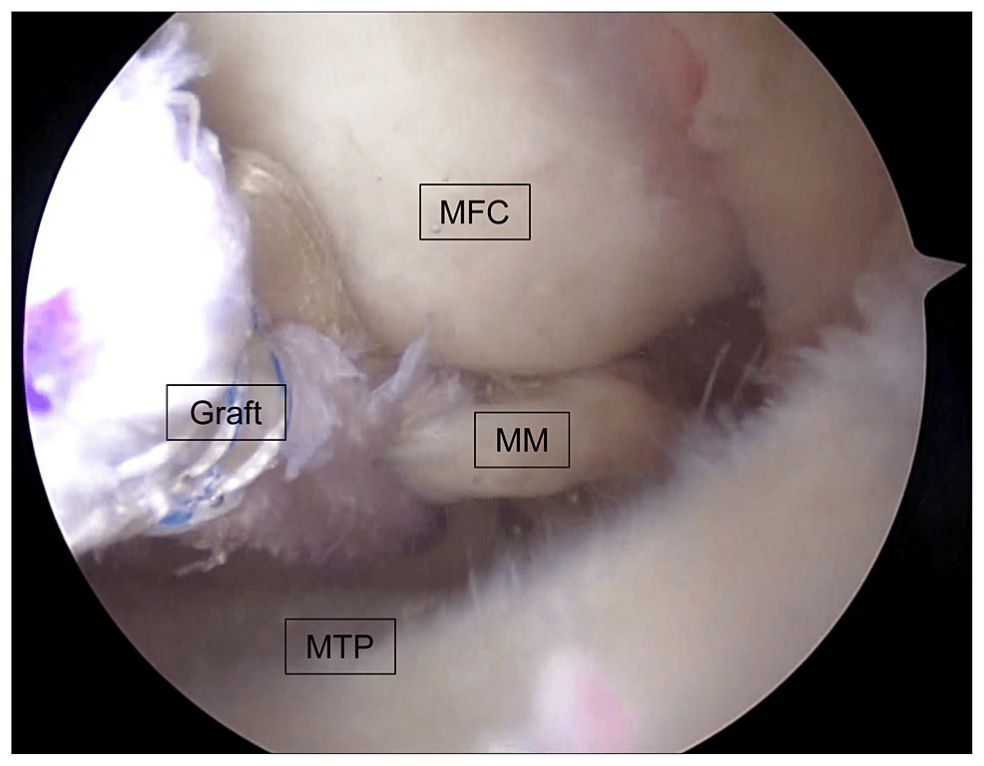
The passing of the graft through the soft tissue tunnel An arthroscopic image of the left knee; the graft was passed through the soft tissue tunnel. MFC: medial femoral condyle; MM: medial meniscus; MTP: medial tibial plateau

Sixth, the graft was passed through the soft tissue tunnel (Figure 5).

**Figure 6:**
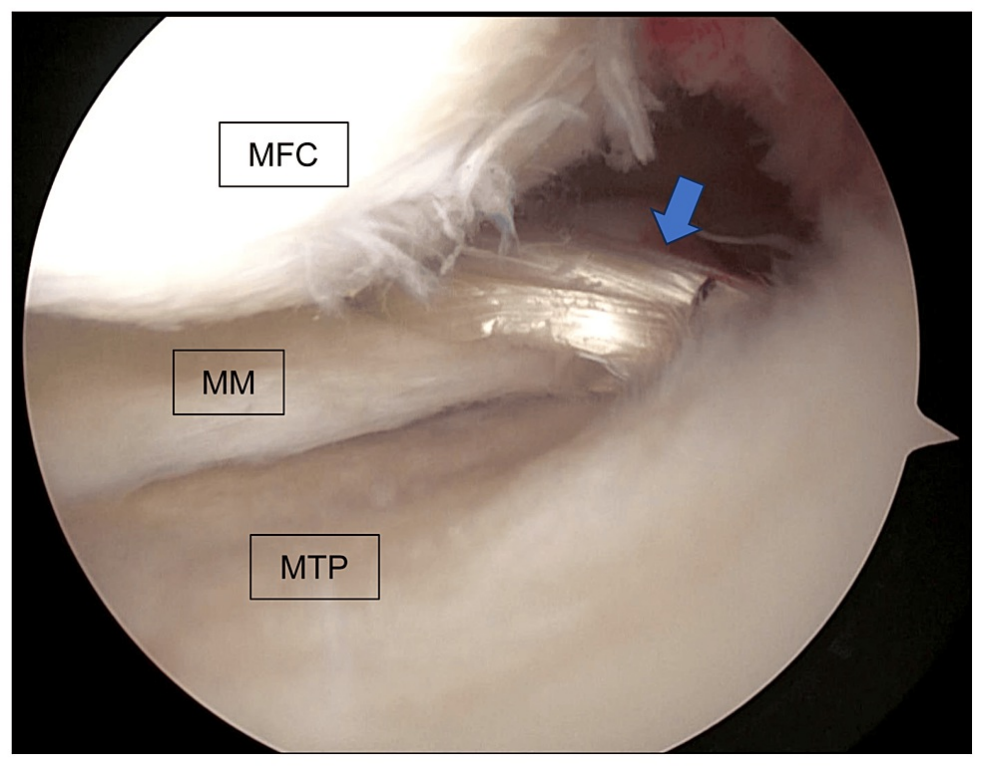
The passage of the graft into the tibial tunnel An arthroscopic image of the left knee; the graft was pulled out into the tibial tunnel and then fixed using an artificial ligament fixture (pull-out button, AI-Medic). MFC: medial femoral condyle; MM: medial meniscus; MTP: medial tibial plateau

**Figure 7:**
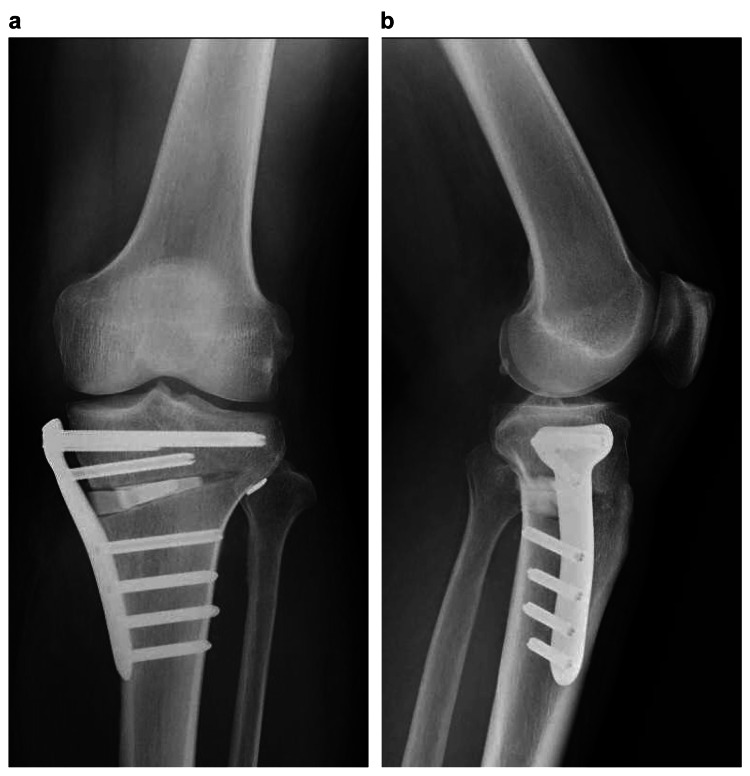
Postoperative plain radiography (a) frontal view; (b) lateral view

Seventh, OWHTO was performed with a target of 62.5 %MA. An artificial bone (OSferion 60, Olympus Terumo Biomaterials, Tokyo, Japan) was inserted into the osteotomy region and fixed using a long locking plate (Tris, Olympus Terumo Biomaterials). Eight, the graft was pulled out into the tibial tunnel and then fixed using an artificial ligament fixture (pull-out button, AI-Medic) (Figures 6-7). 

After two weeks of postoperative non-weight bearing, partial weight-bearing was started, followed by 1/3 weight-bearing at three weeks, 1/2 weight-bearing at four weeks, 2/3 weight-bearing at five weeks, and full loading at six weeks. The range of mobility was 90°-0° until four weeks postoperatively, 120°-0° from four to six weeks, and 130°-0° from six to 12 weeks. Full flexion and squatting were prohibited until three months postoperatively. The patients were allowed to return to sports activities six months after surgery.

Radiological parameters

The FTA was evaluated using the total length of the lower limbs for weight-bearing. The knee was taken in maximum extension, with the patella facing forward. The FTA was defined using the femoral and tibial anatomical axes. The femoral anatomical axis was defined using the line connecting the center of the femoral shaft 10 cm above the intercondylar notch and the intercondylar notch. The tibial anatomical axis was defined as the line connecting the center of the tibial shaft 10 cm below the tibial plateau and the center of the tibial plateau [16].

A lateral view of the knee was taken with the overlapping femoral condyle. The PTS was defined as the angle between the tangent to the MTP and the line perpendicular to the posterior tibial cortex. This method is referred to as the Brazier method, which shows that the posterior tibial cortex is a clear and reliable landmark with minimal systematic error [17].

The %MA was evaluated using the total length of the lower limbs on weight-bearing. It was defined as the length from the medial edge of the tibial plateau to the point of contact between the weight-bearing line and the tibia plateau, divided by the length of the tibial plateau [18]. The weight-bearing line was defined as the line connecting the center of the femoral head and the middle of the ankle joint.

The MME was measured at the intercondylar eminence level on coronal T2-weighted MRI [19, 20]. It was defined as the distance between the medial edge of the tibial plateau and the medial edge of the meniscus. Osteophytes were excluded after determining the medial edge of the tibial plateau [21].

The FTA, PTS, %MA, and MME measurements were evaluated twice, before the index surgery and after the second-look examination.

The FTA, PTS, %MA, and MME were measured twice without patient information by two trained orthopedic surgeons with an interval of at least two weeks. To assess intra- and interobserver reproducibility, these measurements were evaluated using intraclass correlation coefficients (ICCs).

Arthroscopic evaluation

A second-look arthroscopy was performed to remove the OWHTO plate and evaluate the intraarticular joint. The articular cartilage was examined using the ICRS classification from grade 0 to grade IV. Moreover, the articular cartilage during the index surgery and the second-look examination were compared. Based on the ICRS classification, grade 0 was defined as normal cartilage, grade I as the presence of soft indentation stiffness and/or superficial fissures and cracks, grade II as the presence of lesions extending up to 50% of the cartilage depth, grade III as the presence of lesions extending >50% of the cartilage depth, and grade IV as defects on the subchondral bone [22]. The meniscus healing state was defined using the classification of Kim et al. and divided into complete healing (CH), partial healing (PH), and failed healing (FH) where CH was defined as the constructed root showing a nearly normal appearance and tension, PH was defined as good meniscal continuity, with the meniscus lifted slightly on probing, and FH was defined as a complete discontinuity at the reconstructed site [23].

Statistical analysis

Changes in continuous variables (FTA, PTS, %MA, and KOOS) before surgery and at the last follow-up were evaluated using the Wilcoxon signed-rank test. Changes in categorical variables (K-L grade before surgery and during the last follow-up, ICRS grade at the index surgery, and during the second-look examination) were examined using Fisher’s exact test. All statistical analyses were performed using StatFlex version 7 (Artech Co., Ltd., Osaka, Japan) and G*Power version 3.1.9.7 (Universität Kiel, Kiel, Germany). P < 0.05 indicated a statistically significant difference. The sample size was calculated with a power of 81% and an alpha (α) of 0.05 [23]. The required sample size was 12, and 13 patients were finally included in this study.

## Results

In total, 13 knees were analyzed. Table 1 shows the characteristics of the patients. There were 10 (76.9%) women and three (23.1%) men. Their mean age and BMI were 66.8 ± 8.0 years and 26.6 ± 3.4 kg/m^2^, respectively. The study’s mean follow-up duration was 12.8 ± 2.2 months. 

**Table 1:** Characteristics of the patients BMI: body mass index; FU: follow-up; F: female; M: male; Lt: left; Rt: right

Cases	Age (years)	Sex	Site	BMI (kg/m^2^)	FU (months)
1	66	F	Lt	21.5	12
2	63	F	Rt	27.2	12
3	68	F	Lt	28.4	12
4	67	F	Rt	24.4	12
5	78	M	Rt	24.7	12
6	57	F	Lt	34	13
7	76	F	Lt	25	12
8	56	F	Lt	24.6	13
9	61	M	Lt	25.2	20
10	79	M	Lt	31.8	12
11	62	F	Rt	24.8	12
12	72	F	Rt	28.9	12
13	57	F	Lt	24.8	12

The KOOS significantly improved at the last follow-up (P < 0.01) (Tables 2, 3). Based on the FTA and %MA, the varus alignment was predominantly corrected at the last follow-up (P < 0.01) (Tables 2, 3). The MME was increased in nine (62.9%) patients, and the mean MME significantly increased at the last follow-up (P = 0.04). (Tables 2, 3).

**Table 2:** Summary of the clinical, radiological, and second-look results KOOS: Knee Injury and Osteoarthritis Outcome Score; FTA: femorotibial angle; PTS: posterior tibial slope; %MA: percentage mechanical axis; MME: medial meniscus extrusion; K–L: Kellgren–Lawrence; ICRS: International Cartilage Repair Society; MFC: medial femoral condyle; MTP: medial tibial plateau; PO: preoperative; FU: follow-up; CH: complete healing; PH: partial healing; FH: failed healing

Cases	KOOS		FTA (°)		PTS (°)		%MA		MME (mm)		K–L grade		ICRS grade of the MFC			ICRS grade of the MTP		Healing status based on the second-look examination
	PO	Last FU	PO	Last FU	PO	Last FU	PO	Last FU	PO	Last FU	PO	Last FU	PO		Last FU	PO	Last FU	
1	65	82	178.8	172.7	6.4	4	41.8	64	3.5	5.3	1	1	3	3		3	3	CH
2	35	73	175.4	173.9	5.2	2.2	39.8	54.5	4.2	6.4	1	1	2	2		4	4	CH
3	16	88	173.6	172.4	4.0	1.7	48	61.3	5.1	7.0	1	2	4	2		3	1	PH
4	81	87	175.5	173.6	7.2	5.5	30.4	53.8	3.3	7.6	1	1	2	2		2	1	FH
5	29	91	179.3	174.3	9.6	8.0	15.4	55.3	4.6	7.0	1	1	2	2		2	1	PH
6	57	83	173.1	170.1	7.4	4.1	43.7	63.95	3.6	4	1	1	0	0		1	1	CH
7	44	79	178.2	171.9	8.6	7.8	28.8	61.1	3.5	5.5	2	2	3	2		2	2	PH
8	63	91	175.9	172.4	2.3	3.8	38.9	57.1	4.4	4.3	2	2	3	3		2	1	PH
9	48	89	179.5	171.1	11.6	10.6	35	70.4	5.4	2.6	1	1	3	1		1	1	PH
10	78	85	175.2	173.4	9.2	9.6	53.4	57	5.2	5.8	1	1	4	2		2	1	PH
11	53	97	178.3	173.6	3.7	0.7	34	57	5.5	5.5	2	2	3	2		3	2	CH
12	63	91	174.5	171.5	3.1	0.8	467.0	64.2	4.0	3.9	1	1	2	2		1	2	PH
13	43	88	174.3	172.6	6.3	3.4	49.5	57.5	3.9	7.1	1	1	3	2		2	2	PH

**Table 3:** Comparison of the clinical and radiological results before surgery and at the last follow-up KOOS: Knee Injury and Osteoarthritis Outcome Score; FTA: femorotibial angle; PTS: posterior tibial slope; %MA: percentage mechanical axis; MME: medial meniscus extrusion; K–L: Kellgren–Lawrence; ICRS: International Cartilage Repair Society; MFC: medial femoral condyle; MTP: medial tibial plateau Note: Data on KOOS, FTA, PTS, %MA, and MME were expressed as mean ± standard deviation. *P < 0.05, **P < 0.01 ^a^Wilcoxon signed-rank test; ^b^Fisher’s exact test

	Before surgery	Last follow-up	P-value
KOOS	51.9 ± 18.8	86.5 ± 6.2	<0.01**^ a^
FTA	176.3 ± 2.3	172.6 ± 1.2	<0.01**^ a^
PTS	6.5 ± 2.8	4.8 ± 3.3	<0.01**^ a^
%MA	38.9 ± 10.3	59.8 ± 4.9	<0.01**^ a^
MME	4.3 ± 0.8	5.5 ± 1.5	0.04*^ a^
K–L grade (0/1/2/3/4)	0/10/3/0/0/	0/9/4/0/0	0.99^ b ^
ICRS grade of MFC (0/1/2/3/4)	1/0/4/6/2	1/1/9/2/0	0.14^ b^
ICRS grade of MTP (0/1/2/3/4)	0/3/6/3/1	0/7/4/1/1	0.47^ b^

In the second-look examination, the ICRS grade for both MFC and MTP in six (46.2%) patients improved. However, the results did not significantly differ (Tables 2, 3). In terms of meniscus healing, four (30.8%) patients presented with CH, eight (61.5%) with PH, and one (7.7%) with FH. In addition, three patients with improved MME had PH (Tables 2, 3). 

The inter- and intra-observer ICCs of the radiographic and MRI measurements were 0.74-0.99 and 0.84-0.99, respectively. 

## Discussion

This study first reported the use of MMPR-R with OWHTO for MMPRT with varus alignment. The most important finding of this study is that the clinical parameters of the patients predominantly improved. In addition, the healing status rates, including CH and PH, on second-look examination were relatively good at 92.3%. However, the MME increased.

The main treatment options for MMPRT include partial menisectomy, conservative treatment, suture anchor repair, pull-out suture, and OWHTO [5-9, 14, 15]. Meniscus repair has better outcomes than a partial meniscectomy or conservative treatment [24]. Bernard et al. reported that after an average follow-up of 74 months, patients who received conservative treatment or underwent partial meniscectomy were more likely to present with osteoarthritis progression and require arthroplasty than those who underwent meniscus repair. Moreover, arthroplasty was more likely to be required than meniscus repair [24]. Suture anchor repair and pull-out suturing are widely used for meniscus repair, and both procedures have good outcomes [6, 25, 26]. Jung et al. reported an improvement in the Lysholm score from 69.1 to 90.3 with repair using the suture anchor [6]. Furumatsu et al. reported an improvement in the Lysholm score from 61.3 to 86.4 with pull-out suturing using the modified Mason-Allen suture technique [26]. However, Chung et al. reported that the K-L grade progressed in approximately 70% of patients at an average follow-up of 72 months after pull-out suturing [25]. Recently, MMPR-R has attracted significant attention [10, 11]. Li et al. compared MMPR-R and pull-out repair and reported that both the IKDC score and Lysholm score of patients who underwent MMPR-R significantly improved [10]. In addition, Kim et al. revealed that a varus knee alignment of > 5° requires alignment correction [15]. In this study, MMPR-R and OWHTO were performed for MMPRT with varus alignment, resulting in an improvement in the mean KOOS from 51.9 preoperatively to 86.5 postoperatively.

There are several reports on the healing status after meniscus repair in MMPRT using MRI and arthroscopy [5, 6, 23, 27]. Jung et al. evaluated the healing status of 10 patients with suture anchor repair on MRI. Moreover, the authors reported that the CH, PH, and FH rates of the patients were 50%, 40%, and 10%, respectively [6]. Moon et al. evaluated the healing status of patients who underwent pull-out suturing on MRI. Results showed that the CH, PH, and FH rates of the patients were 90%, 10%, and 0%, respectively [27]. By contrast, Seo et al. evaluated the healing status of 11 patients who underwent pull-out suturing using arthroscopy. They reported that the CH, PH, and FH rates of the patients were 0%, 82%, and 18%, respectively [5]. Further, the CH, PH, and FH rates of patients who underwent surgery using the remodified Manson-Allen suture technique and OWHTO were 64.2%, 29.4%, and 5.9%, respectively [23]. Recently, Wang et al. first evaluated the healing status of patients who underwent MMPR reconstruction on MRI. They reported that the CH, PH, and FH rates of reconstructions were 82.8%, 10.3%, and 6.9%, respectively [10]. However, the healing status on the second-look examination was not reported [10]. In this study, MMPR-R and OWHTO were performed. The outcomes were evaluated using arthroscopy, with CH, PH, and FH rates of 30.8%, 61.5%, and 7.7%, respectively. In addition, there was no significant improvement in the ICRS grade of MFC. However, eight (61.5%) cases of ICRS classification G3/4 improved to two (15.4%) cases.

A medial meniscus posterior root tear leads to medial meniscus hoop collapse and increased MME [1, 2]. Hence, the contact pressure on the medial side of the tibiofemoral increases, causing the progression of knee osteoarthritis [3, 4]. Therefore, it is important to repair these hoops and further improve MME [28]. Jung et al. reported that the mean MME decreased from 3.9 mm preoperatively to 3.5 mm postoperatively after suture anchor repair [6]. Moon et al. reported an increase in the mean MME from 3.6 mm preoperatively to 5.0 mm after pull-out repair [27]. In the study by Kim et al., the mean MME increased from 3.0 mm preoperatively to 3.1 mm postoperatively using the remodified Manson-Allen suture technique and OWHTO [23]. Furthermore, a recent study has reported that MMPRT centralization is useful for improving MME on MMPRT [29]. Mochizuki et al. showed that the average MME decreased significantly from 4.8 to 2.7 mm with suture anchor repair combined with centralization [29]. This phenomenon resulted in a significant increase in the average MME from 4.3 to 5.5 mm. Furthermore, the patients with improved MME had PH on the second-look examination. Meanwhile, the MME of other patients with CH increased. Ishikawa et al. reported the use of a technique for improving MMPR-R with pull-out sutures and assisted sutures. In addition to MMPR-R, additional procedures such as pull-out suturing and centralization can have better outcomes.

The current study reported the outcomes of MMPR-R and OWHTO for MMPRT with varus alignment. The KOOS led to an improvement from 51.9 preoperatively to 86.5 at the last follow-up. The healing rate for combined CH and PH was 92.3%, and the hoops were well reconstructed. By contrast, the mean MME increased from 4.3 mm preoperatively to 5.5 mm at the last follow-up. Moreover, the addition of centralization and pull-out sutures could improve MME.

The current study has several limitations. First, it was retrospective in nature. Thus, patient selection bias might have existed. Second, a control group was not included. Both MMPR-R and OWHTO had good outcomes. Therefore, whether the combined use of MMPR-R and OWHTO had good outcomes was not validated. Third, the sample size was small. Hence, future studies with larger prospective sample sizes must be performed. Finally, the follow-up period was short. Thus, a longer follow-up is required in future studies.

## Conclusions

We described 13 cases of MMPRT with varus knee alignment treated with MMPR-R and OWHTO to identify an optimal MMPRT treatment. Medial meniscus posterior root tear with varus knee alignment was significantly improved with MMPR-R and OWHTO. However, the MME and meniscus healing were not satisfactory. 
